# 4-(Cyano­meth­yl)anilinium 4-methyl­benzene­sulfonate monohydrate

**DOI:** 10.1107/S1600536810020180

**Published:** 2010-06-05

**Authors:** Jin Rui Lin

**Affiliations:** a Ordered Matter Science Research Center, Southeast University, Nanjing 210096, People’s Republic of China

## Abstract

In the title salt, C_8_H_9_N_2_^+^·C_7_H_7_O_3_S^−^·H_2_O, the dihedral angle between the cation and anion benzene rings is 50.1 (4)°. In the cation, the cyano­methyl group is twisted from the plane of the benzene ring [C—C—C—N = −86 (12)°]. In the crystal, the cations, anions and water mol­ecules are linked by N—H⋯O and O—H⋯O hydrogen bonds, forming a chain along the *c* axis.

## Related literature

For phase transition materials and metal-organic coordination compounds, see: Zhang *et al.* (2009 ▶); Li *et al.* (2008 ▶); Liu *et al.* (2005 ▶). For bond-length data, see: Allen *et al.* (1987 ▶).
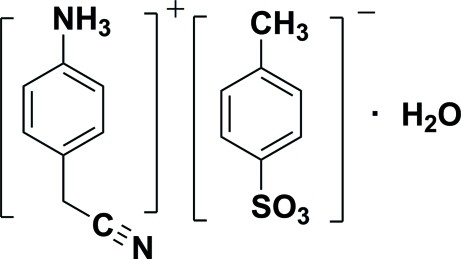


## Experimental

### 

#### Crystal data

C_8_H_9_N_2_^+^·C_7_H_7_O_3_S^−^·H_2_O
*M* *_r_* = 322.38
Tetragonal, 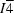

*a* = 22.931 (2) Å
*c* = 5.946 (2) Å
*V* = 3126.6 (11) Å^3^
*Z* = 8
Mo *K*α radiation
μ = 0.23 mm^−1^
*T* = 293 K
0.45 × 0.40 × 0.25 mm

#### Data collection

Rigaku SCXmini diffractometer
Absorption correction: multi-scan (*CrystalClear*; Rigaku, 2005 ▶) *T*_min_ = 0.903, *T*_max_ = 0.945
15184 measured reflections
3089 independent reflections
2723 reflections with *I* > 2σ(*I*)
*R*_int_ = 0.068

#### Refinement

*R*[*F* ^2^ > 2σ(*F* ^2^)] = 0.072
*wR*(*F* ^2^) = 0.196
*S* = 1.05
3089 reflections
200 parameters
5 restraints
H-atom parameters constrained
Δρ_max_ = 0.54 e Å^−3^
Δρ_min_ = −0.24 e Å^−3^
Absolute structure: Flack (1983 ▶), 1383 Friedel pairs
Flack parameter: 0.05 (16)

### 

Data collection: *CrystalClear* (Rigaku, 2005 ▶); cell refinement: *CrystalClear*; data reduction: *CrystalClear*; program(s) used to solve structure: *SHELXS97* (Sheldrick, 2008 ▶); program(s) used to refine structure: *SHELXL97* (Sheldrick, 2008 ▶); molecular graphics: *SHELXTL* (Sheldrick, 2008 ▶); software used to prepare material for publication: *PRPKAPPA* (Ferguson, 1999 ▶).

## Supplementary Material

Crystal structure: contains datablocks global, I. DOI: 10.1107/S1600536810020180/jj2033sup1.cif

Structure factors: contains datablocks I. DOI: 10.1107/S1600536810020180/jj2033Isup2.hkl

Additional supplementary materials:  crystallographic information; 3D view; checkCIF report

## Figures and Tables

**Table 1:** Hydrogen-bond geometry (Å, °)

*D*—H⋯*A*	*D*—H	H⋯*A*	*D*⋯*A*	*D*—H⋯*A*
O4—H4*D*⋯O3	0.85	1.90	2.746 (7)	179
N1—H1*A*⋯O3^i^	0.89	2.09	2.886 (6)	148
N1—H1*B*⋯O1^ii^	0.89	2.11	2.850 (6)	140
N1—H1*C*⋯O4	0.89	2.35	2.972 (6)	127

Symmetry codes: (i) 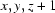; (ii) 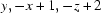.

**Fig. 1.**
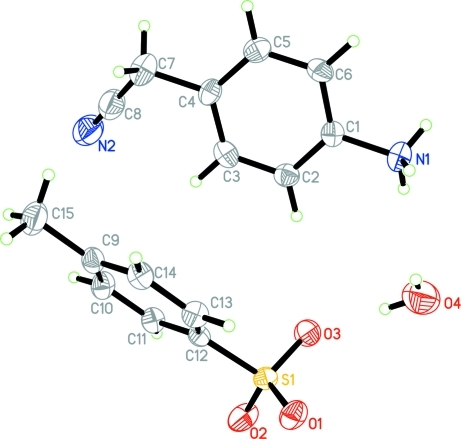
The molecular structure of the title compound, showing the atomic numbering scheme. Displacement ellipsoids are drawn at the 30% probability level.

**Fig. 2.**
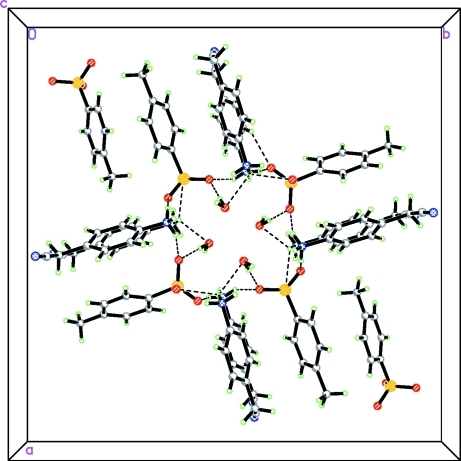
A view of the packing of the title compound, with stacking along the c axis. Dashed lines indicate N—H···O and O—H···O hydrogen bonds.

## supplementary crystallographic information

### Comment

The title compound, (I), is a continuation of our study of phase transition materials, which include organic ligands (Li *et al.*, 2008), metal-organic coordination compounds (Zhang*et al.*, 2009) and the dielectric constant of 4-(cyanomethyl)anilinium 4-methylbenzenesulfonate as a function of temperature. Our study indicating that the permittivity is temperature-independent (dielectric constant equals 7.6 to 14.1), suggests that there may be no distinct phase transition in (I) within the measured temperature range.

The asymmetric unit of the title compound (Fig.1),contains 4-(cyanomethyl)anilinium cations, 4-methylbenzenesulfonate anions and water molecules in the stoichiometric ratio of 1:1:1.  The dihedral angle between the two cation-anion benzene rings is 50.1 (4)°.  In the cation, the cyanomethyl group is twisted from the plane of the benzene ring (C4/C7/C8/N2 = -86 (12)°) and the methyl group is planar with the ring. In the anion, both the sylfonyl and methyl groups are planar with the benxene ring.  Bond distances (Allen *et al.*, 1987) and angles are in normal ranges.  In the crystal structure (Fig.2), cations, anions and water molecules are linked by intermolecular N—H···O and O—H···O hydrogen bonds, forming a one-dimensional chain along the *c* axis, assisting crystal packing.

### Experimental

2-(4-aminophenyl)acetonitrile was prepared from 2-(4-nitrophenyl)acetonitrile according to the reported method (Liu Y *et al.*, 2005). Single crystals of 4-(cyanomethyl)anilinium 4-methylbenzenesulfonate were prepared by slow evaporation at room temperature of an equuimolar methanol-water solution for 10 h.

### Refinement

All the hydrogen atoms could have been discerned in the difference electron density map, nevertheless, all the H atoms attached to the carbon atoms were constrained in a riding motion approximation. C_aryl_—H = 0.93 Å, with U_ĩso_(H) = 1.2*U*_eq_(C). C_methyl_—H = 0.96 Å, with *U*_iso_(H)=1.5*U*_eq_(C). N—H = 0.89 Å, U_ĩso_(H) = 1.5*U*_eq_(N). The hydroxyl hydrogen were placed at ideal positions and refined using a 'rotating' model for hydroxyl H atom with *U*_iso_(H) = 1.5 *U*_eq_ (O).

### Crystal data

**Table:**

C_8_H_9_N_2_^+^·C_7_H_7_O_3_S^−^·H_2_O	*D*_x_ = 1.370 Mg m^−^^3^
*M**_r_* = 322.38	Mo *K*α radiation, λ = 0.71073 Å
Tetragonal, *I*4	Cell parameters from 5655 reflections
Hall symbol: I -4	θ = 3.5–27.5°
*a* = 22.931 (2) Å	µ = 0.23 mm^−^^1^
*c* = 5.946 (2) Å	*T* = 293 K
*V* = 3126.6 (11) Å^3^	Prism, orange
*Z* = 8	0.45 × 0.40 × 0.25 mm
*F*(000) = 1360	

### Data collection

**Table:**

Rigaku SCXmini diffractometer	3089 independent reflections
Radiation source: fine-focus sealed tube	2723 reflections with *I* > 2σ(*I*)
graphite	*R*_int_ = 0.068
Detector resolution: 13.6612 pixels mm^-1^	θ_max_ = 26.0°, θ_min_ = 3.5°
CCD_Profile_fitting scans	*h* = −28→28
Absorption correction: multi-scan (*CrystalClear*; Rigaku, 2005)	*k* = −28→28
*T*_min_ = 0.903, *T*_max_ = 0.945	*l* = −7→7
15184 measured reflections	

### Refinement

**Table:**

Refinement on *F*^2^	Secondary atom site location: difference Fourier map
Least-squares matrix: full	Hydrogen site location: inferred from neighbouring sites
*R*[*F*^2^ > 2σ(*F*^2^)] = 0.072	H-atom parameters constrained
*wR*(*F*^2^) = 0.196	*w* = 1/[σ^2^(*F*_o_^2^) + (0.120*P*)^2^ + 1.4624*P*] where *P* = (*F*_o_^2^ + 2*F*_c_^2^)/3
*S* = 1.05	(Δ/σ)_max_ = 0.001
3089 reflections	Δρ_max_ = 0.54 e Å^−^^3^
200 parameters	Δρ_min_ = −0.24 e Å^−^^3^
5 restraints	Absolute structure: Flack (1983), 1383 Friedel pairs
Primary atom site location: structure-invariant direct methods	Flack parameter: 0.05 (16)

### Special details

**Table:**

Geometry. All e.s.d.'s (except the e.s.d. in the dihedral angle between two l.s. planes) are estimated using the full covariance matrix. The cell e.s.d.'s are taken into account individually in the estimation of e.s.d.'s in distances, angles and torsion angles; correlations between e.s.d.'s in cell parameters are only used when they are defined by crystal symmetry. An approximate (isotropic) treatment of cell e.s.d.'s is used for estimating e.s.d.'s involving l.s. planes.
Refinement. Refinement of *F*^2^ against ALL reflections. The weighted *R*-factor *wR* and goodness of fit *S* are based on *F*^2^, conventional *R*-factors *R* are based on *F*, with *F* set to zero for negative *F*^2^. The threshold expression of *F*^2^ > σ(*F*^2^) is used only for calculating *R*-factors(gt) *etc*. and is not relevant to the choice of reflections for refinement. *R*-factors based on *F*^2^ are statistically about twice as large as those based on *F*, and *R*- factors based on ALL data will be even larger.

### Fractional atomic coordinates and isotropic or equivalent isotropic displacement parameters (Å^2^)

**Table:**

	*x*	*y*	*z*	*U*_iso_*/*U*_eq_	
C9	0.80554 (19)	0.68688 (19)	0.6159 (9)	0.0451 (10)	
C10	0.7993 (2)	0.6601 (2)	0.4099 (9)	0.0509 (12)	
H10A	0.8321	0.6557	0.3195	0.061*	
C11	0.74716 (19)	0.6397 (2)	0.3328 (8)	0.0444 (11)	
H11A	0.7449	0.6211	0.1942	0.053*	
C12	0.69775 (19)	0.64693 (18)	0.4622 (7)	0.0368 (9)	
C13	0.7012 (2)	0.6746 (2)	0.6699 (8)	0.0451 (11)	
H13A	0.6680	0.6802	0.7575	0.054*	
C14	0.7554 (2)	0.6937 (2)	0.7428 (9)	0.0486 (11)	
H14A	0.7581	0.7118	0.8823	0.058*	
C15	0.8629 (3)	0.7090 (3)	0.7022 (12)	0.0718 (17)	
H15A	0.8929	0.7011	0.5937	0.086*	
H15B	0.8722	0.6898	0.8412	0.086*	
H15C	0.8604	0.7503	0.7269	0.086*	
O1	0.58469 (14)	0.65351 (15)	0.4733 (6)	0.0567 (10)	
O2	0.62991 (15)	0.62234 (19)	0.1289 (6)	0.0679 (10)	
O3	0.62786 (15)	0.55881 (15)	0.4448 (7)	0.0608 (10)	
O4	0.5601 (2)	0.5210 (3)	0.7965 (10)	0.112 (2)	
H4D	0.5808	0.5324	0.6864	0.168*	
H4B	0.5707	0.5387	0.9153	0.168*	
S1	0.62993 (5)	0.61860 (5)	0.37106 (18)	0.0416 (3)	
C1	0.7116 (2)	0.49056 (19)	1.0648 (8)	0.0409 (10)	
C2	0.7262 (2)	0.51689 (19)	0.8664 (9)	0.0468 (10)	
H2B	0.6985	0.5227	0.7546	0.056*	
C3	0.7830 (2)	0.5346 (2)	0.8365 (9)	0.0512 (12)	
H3B	0.7934	0.5536	0.7042	0.061*	
C4	0.8251 (2)	0.5248 (2)	0.9986 (9)	0.0451 (11)	
C5	0.8087 (2)	0.4970 (2)	1.1943 (9)	0.0499 (12)	
H5A	0.8365	0.4896	1.3044	0.060*	
C6	0.7516 (2)	0.4798 (2)	1.2302 (8)	0.0495 (11)	
H6A	0.7407	0.4614	1.3631	0.059*	
C7	0.8871 (2)	0.5456 (3)	0.9678 (10)	0.0616 (15)	
H7A	0.8908	0.5843	1.0322	0.074*	
H7B	0.9130	0.5199	1.0501	0.074*	
C8	0.9054 (3)	0.5476 (3)	0.7358 (13)	0.0763 (18)	
N1	0.65068 (16)	0.47274 (18)	1.1017 (8)	0.0531 (10)	
H1A	0.6316	0.5013	1.1716	0.080*	
H1B	0.6499	0.4407	1.1861	0.080*	
H1C	0.6338	0.4655	0.9699	0.080*	
N2	0.9217 (3)	0.5462 (3)	0.5409 (11)	0.0937 (19)	

### Atomic displacement parameters (Å^2^)

**Table:**

	*U*^11^	*U*^22^	*U*^33^	*U*^12^	*U*^13^	*U*^23^
C9	0.045 (2)	0.037 (2)	0.053 (3)	−0.0032 (18)	0.004 (2)	0.001 (2)
C10	0.043 (2)	0.056 (3)	0.054 (3)	0.001 (2)	0.015 (2)	−0.003 (2)
C11	0.048 (3)	0.045 (2)	0.041 (2)	0.001 (2)	0.009 (2)	−0.006 (2)
C12	0.043 (2)	0.031 (2)	0.036 (2)	0.0017 (18)	0.0014 (18)	0.0012 (17)
C13	0.050 (3)	0.046 (2)	0.039 (3)	0.004 (2)	0.005 (2)	−0.007 (2)
C14	0.056 (3)	0.043 (2)	0.048 (3)	0.000 (2)	0.005 (2)	−0.003 (2)
C15	0.056 (3)	0.069 (4)	0.091 (5)	−0.015 (3)	−0.005 (3)	−0.005 (3)
O1	0.0433 (18)	0.059 (2)	0.068 (2)	0.0101 (16)	0.0096 (17)	−0.0105 (18)
O2	0.051 (2)	0.111 (3)	0.0417 (18)	0.009 (2)	0.0004 (18)	−0.007 (2)
O3	0.059 (2)	0.0404 (18)	0.083 (3)	−0.0032 (16)	−0.0072 (19)	0.0012 (17)
O4	0.107 (4)	0.118 (4)	0.110 (5)	0.026 (3)	0.014 (3)	0.029 (4)
S1	0.0416 (6)	0.0415 (6)	0.0416 (5)	0.0043 (5)	0.0002 (5)	−0.0030 (5)
C1	0.041 (2)	0.037 (2)	0.044 (3)	0.0030 (18)	0.0073 (19)	−0.0069 (19)
C2	0.052 (3)	0.042 (2)	0.047 (3)	0.001 (2)	−0.005 (2)	0.000 (2)
C3	0.063 (3)	0.045 (3)	0.046 (3)	−0.003 (2)	0.008 (2)	0.001 (2)
C4	0.040 (2)	0.045 (3)	0.050 (3)	−0.0037 (19)	0.001 (2)	−0.012 (2)
C5	0.049 (3)	0.053 (3)	0.048 (3)	0.003 (2)	−0.005 (2)	−0.008 (2)
C6	0.060 (3)	0.052 (3)	0.036 (2)	0.000 (2)	−0.001 (2)	0.006 (2)
C7	0.052 (3)	0.070 (4)	0.062 (3)	−0.006 (3)	0.005 (3)	−0.027 (3)
C8	0.056 (4)	0.095 (5)	0.078 (5)	−0.012 (3)	0.001 (3)	0.003 (4)
N1	0.042 (2)	0.058 (2)	0.059 (3)	−0.0061 (18)	0.005 (2)	−0.009 (2)
N2	0.077 (4)	0.124 (5)	0.080 (4)	−0.016 (4)	−0.012 (3)	0.005 (4)

### Geometric parameters (Å, °)

**Table:**

C9—C10	1.378 (7)	C1—C2	1.367 (7)
C9—C14	1.384 (7)	C1—C6	1.367 (7)
C9—C15	1.500 (7)	C1—N1	1.473 (6)
C10—C11	1.363 (7)	C2—C3	1.376 (7)
C10—H10A	0.9300	C2—H2B	0.9300
C11—C12	1.379 (6)	C3—C4	1.383 (7)
C11—H11A	0.9300	C3—H3B	0.9300
C12—C13	1.391 (6)	C4—C5	1.380 (7)
C12—S1	1.771 (4)	C4—C7	1.511 (7)
C13—C14	1.388 (7)	C5—C6	1.384 (7)
C13—H13A	0.9300	C5—H5A	0.9300
C14—H14A	0.9300	C6—H6A	0.9300
C15—H15A	0.9600	C7—C8	1.442 (9)
C15—H15B	0.9600	C7—H7A	0.9700
C15—H15C	0.9600	C7—H7B	0.9700
O1—S1	1.445 (3)	C8—N2	1.218 (9)
O2—S1	1.443 (4)	N1—H1A	0.8900
O3—S1	1.440 (4)	N1—H1B	0.8900
O4—H4D	0.8500	N1—H1C	0.8900
O4—H4B	0.8499		
			
C10—C9—C14	116.6 (4)	C2—C1—C6	122.5 (4)
C10—C9—C15	123.1 (5)	C2—C1—N1	118.9 (4)
C14—C9—C15	120.2 (5)	C6—C1—N1	118.6 (4)
C11—C10—C9	122.9 (4)	C1—C2—C3	118.3 (5)
C11—C10—H10A	118.5	C1—C2—H2B	120.9
C9—C10—H10A	118.5	C3—C2—H2B	120.9
C10—C11—C12	119.4 (5)	C2—C3—C4	121.5 (5)
C10—C11—H11A	120.3	C2—C3—H3B	119.3
C12—C11—H11A	120.3	C4—C3—H3B	119.3
C11—C12—C13	120.3 (4)	C3—C4—C5	118.2 (4)
C11—C12—S1	120.4 (3)	C3—C4—C7	121.4 (5)
C13—C12—S1	119.3 (3)	C5—C4—C7	120.4 (5)
C14—C13—C12	118.2 (4)	C6—C5—C4	121.4 (5)
C14—C13—H13A	120.9	C6—C5—H5A	119.3
C12—C13—H13A	120.9	C4—C5—H5A	119.3
C9—C14—C13	122.6 (5)	C1—C6—C5	118.1 (4)
C9—C14—H14A	118.7	C1—C6—H6A	120.9
C13—C14—H14A	118.7	C5—C6—H6A	120.9
C9—C15—H15A	109.5	C8—C7—C4	113.6 (5)
C9—C15—H15B	109.5	C8—C7—H7A	108.9
H15A—C15—H15B	109.5	C4—C7—H7A	108.9
C9—C15—H15C	109.5	C8—C7—H7B	108.9
H15A—C15—H15C	109.5	C4—C7—H7B	108.9
H15B—C15—H15C	109.5	H7A—C7—H7B	107.7
H4D—O4—H4B	109.5	N2—C8—C7	176.5 (9)
O3—S1—O2	111.1 (3)	C1—N1—H1A	109.5
O3—S1—O1	112.1 (2)	C1—N1—H1B	109.5
O2—S1—O1	112.8 (2)	H1A—N1—H1B	109.5
O3—S1—C12	106.6 (2)	C1—N1—H1C	109.5
O2—S1—C12	106.5 (2)	H1A—N1—H1C	109.5
O1—S1—C12	107.4 (2)	H1B—N1—H1C	109.5
			
C14—C9—C10—C11	−1.5 (7)	C13—C12—S1—O1	28.4 (4)
C15—C9—C10—C11	179.5 (5)	C6—C1—C2—C3	−2.0 (7)
C9—C10—C11—C12	1.5 (8)	N1—C1—C2—C3	178.4 (4)
C10—C11—C12—C13	−0.2 (7)	C1—C2—C3—C4	1.9 (7)
C10—C11—C12—S1	−177.7 (4)	C2—C3—C4—C5	−0.5 (7)
C11—C12—C13—C14	−0.9 (7)	C2—C3—C4—C7	−178.5 (5)
S1—C12—C13—C14	176.7 (4)	C3—C4—C5—C6	−0.8 (7)
C10—C9—C14—C13	0.3 (7)	C7—C4—C5—C6	177.2 (5)
C15—C9—C14—C13	179.3 (5)	C2—C1—C6—C5	0.8 (7)
C12—C13—C14—C9	0.8 (7)	N1—C1—C6—C5	−179.6 (4)
C11—C12—S1—O3	85.7 (4)	C4—C5—C6—C1	0.7 (7)
C13—C12—S1—O3	−91.8 (4)	C3—C4—C7—C8	−30.1 (8)
C11—C12—S1—O2	−33.0 (4)	C5—C4—C7—C8	152.0 (6)
C13—C12—S1—O2	149.5 (4)	C4—C7—C8—N2	−86 (12)
C11—C12—S1—O1	−154.0 (4)		

### Hydrogen-bond geometry (Å, °)

**Table:**

*D*—H···*A*	*D*—H	H···*A*	*D*···*A*	*D*—H···*A*
O4—H4D···O3	0.85	1.90	2.746 (7)	179
N1—H1A···O3^i^	0.89	2.09	2.886 (6)	148
N1—H1B···O1^ii^	0.89	2.11	2.850 (6)	140
N1—H1C···O4	0.89	2.35	2.972 (6)	127

Symmetry codes: (i) *x*, *y*, *z*+1; (ii) *y*, −*x*+1, −*z*+2.
